# Optimizing Interfacial Bonding of Additive Manufactured Resin via Combined Surface Treatments: An In Vitro Study on 3D-Printed Fixed Lingual Retainers

**DOI:** 10.3390/jfb17100501

**Published:** 2026-10-03

**Authors:** Pyi Phyo Win, I-Ying Huang, Daniel De-Shing Chen, Szu-Yu Lai, Yuichi Mine, Tong-Hsien Chow, I-Ta Lee, Chien-Fu Tseng, Tzu-Yu Peng

**Affiliations:** 1 School of Dentistry, College of Oral Medicine, Taipei Medical University, Taipei 110301, Taiwan; 2 Department of Oral Hygiene, College of Dental Medicine, Kaohsiung Medical University, Kaohsiung 807378, Taiwan; 3 Department of Dentistry, Taoyuan General Hospital, Ministry of Health and Welfare, Taoyuan 330004, Taiwan; 4 Research Center for Tooth Bank and Dental Stem Cell Technology, College of Oral Medicine, Taipei Medical University, Taipei 110301, Taiwan; 5 Division of Orthodontics, Department of Oral Medicine, Taipei Medical University Hospital, Taipei 110301, Taiwan; 6 Research Center of Precision Biomedical Implants, College of Oral Medicine, Taipei Medical University, Taipei 110301, Taiwan; 7 Project Research Center for Integrating Digital Dentistry, Hiroshima University, Hiroshima 734-8553, Japan; 8 Department of Medical Systems Engineering, Graduate School of Biomedical and Health Sciences, Hiroshima University, Hiroshima 734-8553, Japan; 9 Department of Sports Science, R.O.C. Military Academy, Kaohsiung 830208, Taiwan; 10 Graduate Institute of Clinical Dentistry, School of Dentistry, College of Medicine, National Taiwan University, Taipei 100229, Taiwan; 11 Interdisciplinary Undergraduate Program in Intelligent Healthcare, College of Interdisciplinary Studies, Taipei Medical University, Taipei 110301, Taiwan

**Keywords:** 3D-printed resin, dental polymers, adhesive bonding, surface modification, airborne-particle abrasion, non-thermal handheld plasma, universal adhesive, resin-matrix penetrating primer

## Abstract

Fixed lingual retainers fabricated from 3D-printed resin (3DR) offer the benefits of geometric freedom; however, conventional approaches frequently rely on encapsulation or oversized retention pads to compensate for limited adhesion, increasing bulk and undermining these advantages. This study evaluated an interface-only bonding protocol using combinations of mechanical and chemical surface treatments for 3DR. Specimens were assigned to five groups: control; airborne-particle abrasion (APA) followed by universal adhesive (UA); APA followed by resin-matrix-penetrating primer (RMPP); non-thermal handheld plasma (NTHP) followed by UA; and NTHP followed by RMPP. Surface roughness, wettability, and chemical composition were characterized using contact profilometry, water-contact angle measurements, and attenuated total reflectance Fourier-transform infrared spectroscopy, respectively. Shear bond strength (SBS) and failure modes were evaluated before and after 10,000 thermal cycles. APA-containing protocols significantly increased surface roughness, whereas NTHP-containing protocols improved wettability without detectable topographical changes. Distinct chemical signals associated with urethane dimethacrylate and 10-methacryloyloxydecyl dihydrogen phosphate were identified in the RMPP- and UA-treated groups, respectively. Pre-aging SBS was comparable among groups, whereas APA followed by RMPP produced the highest post-aging SBS, and UA-containing groups showed the lowest values. These findings suggest that the combination of APA and RMPP improves resistance to in vitro hydrothermal degradation, although intraoral durability of interface-only 3DR retainers requires further clinical validation.

## 1. Introduction

The advent of computer-aided design and computer-aided manufacturing (CAD/CAM) has ushered in a new era of precision and customization in dentistry [1,2]. Within this digital workflow, additive manufacturing (AM) or three-dimensional (3D) printing offers distinct advantages, such as reduced material waste and unique capacity to fabricate complex geometries over traditional subtractive milling [3]. Among AM technologies, digital light processing (DLP) printing enables rapid, full-layer curing and delivers exceptional edge sharpness through precise digital pixel control [4]. Recently, 3D-printed resins (3DRs), which were initially developed for permanent crowns, have emerged as a promising alternative material for broader clinical applications. Their proven long-term intraoral stability and favorable mechanical properties make them suitable for the fabrication of fixed lingual retainers (FLRs) [5,6]. However, achieving durable adhesion to additively manufactured photopolymer resins remain challenging, as untreated 3D-printed surfaces often exhibit inferior bond strength compared with surface-treated specimens [7]. During photopolymerization, atmospheric oxygen acts as a radical scavenger, forming an oxygen-inhibited layer containing unreacted monomers that reduces surface polymerization and compromises surface properties [8]. Subsequent post-curing increases monomer conversion and promotes more complete polymerization throughout the printed structure. Consequently, surface engineering is a critical prerequisite for enhancing surface roughness, surface energy, and wettability, thereby facilitating reliable adhesive bonding [7].

Maintaining orthodontic treatment outcomes remains a persistent challenge as posttreatment relapse threatens to undermine significant investments in time and effort made by both clinicians and patients [9]. FLRs are frequently indispensable in mitigating this risk, particularly when long-term patient compliance is a concern [10]. For decades, multistranded stainless steel wires have served as the clinical gold standard for fixed retention [11]. However, they possess inherent limitations: manual fabrication is a technique-sensitive process that struggles to achieve a perfectly passive fit and frequently leads to unwanted tooth movement [12,13]. Furthermore, the color of these wires can be unesthetic, and their metallic composition can trigger adverse reactions in patients with nickel hypersensitivity, thereby generating substantial artifacts that compromise the diagnostic quality of magnetic resonance imaging [14,15].

**Figure 1:**
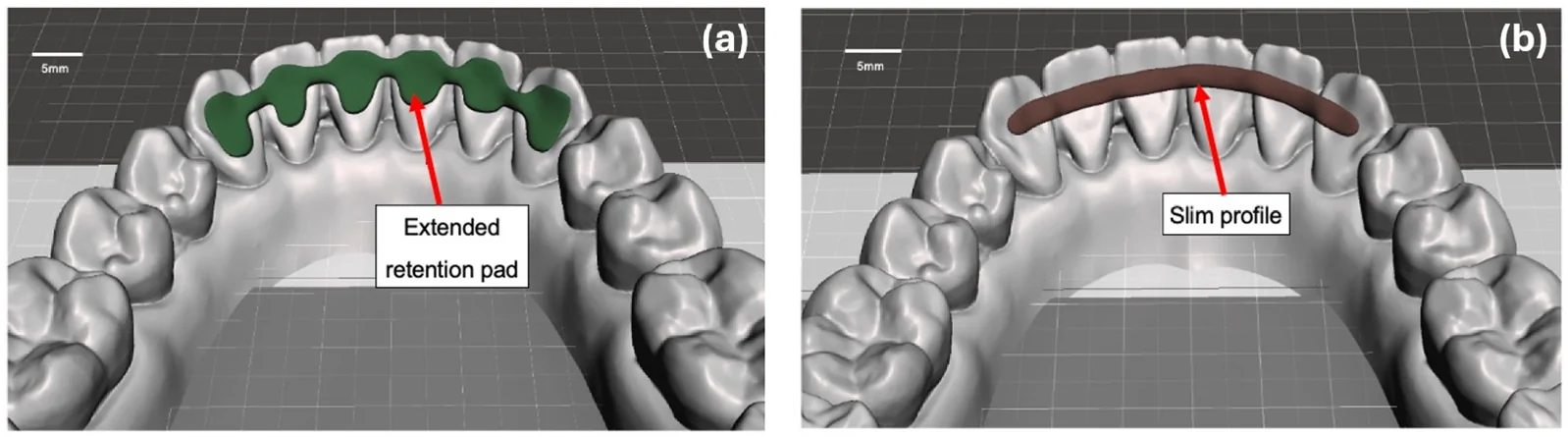
Virtual models of 3D-printed fixed lingual retainers on a mandibular arch: (**a**) design utilizing retention pad for enhanced surface contact (green); (**b**) minimalist, interface-only design (brown). Scale bar = 5 mm. The background viewport grid consists of 5 × 5 mm squares and is provided as a dimensional reference for the 3D perspective rendering.

Despite the potential of 3DRs to resolve these issues, current research frequently replicates traditional “encapsulation” methods which trap the appliance within bulk resin composite or utilize oversized retention pads to compensate for adhesive limitations [16,17,18,19]. Although these features increase the bond strength, they negate the clinical benefit of 3D printing, which is a streamlined, hygienic, and low-profile appliance. This study, therefore, evaluates an ‘interface-only’ bonding protocol that eliminates bulk coverage and oversized pads while placing the entire burden of durability on a minimalist adhesive interface. A schematic comparison highlighting the structural differences between extended retention pads and the proposed interface-only FLR designs is illustrated in Figure 1.

The translation of 3DRs to this minimalist design introduces a critical challenge: achieving a durable bond to a highly cross-linked polymer network [20,21]. Conventional surface-modification strategies include airborne-particle abrasion (APA) and universal adhesives (UAs) containing 10-methacryloyloxydecyl dihydrogen phosphate (10-MDP) [7,22,23,24,25]. However, these methods exhibit several limitations. APA is limited by its high operator dependency, as variations in application parameters may compromise the surface consistency and increase the risk of structural defects, especially with the treatment of small and delicate appliances, such as FLRs [26], whereas the chemical bonding efficacy of 10-MDP seems limited to densely cross-linked polymers without prior mechanical roughening [21].

Therefore, several alternative strategies have been developed. Nonthermal handheld plasma (NTHP) offers a minimally invasive approach that increases surface energy and wettability without inducing microstructural damage [27,28,29]. Resin matrix-penetrating primers (RMPPs) represent a specialized chemical strategy. Originally formulated to condition high-strength, highly cross-linked CAD/CAM composite blocks, these primers contain solvents and functional monomers designed to penetrate and co-polymerize with the resin matrix, and thereby offer a potential pathway for enhanced chemical interaction with structurally analogous 3D-printed materials [30,31]. Notably, methyl methacrylate (MMA)-containing primers can achieve higher bond strengths than conventional silane agents without APA, and thereby reduce the risk of structural damage [32].

To identify existing evidence, a structured search of electronic databases (PubMed and Web of Science) was performed through March 2026. Although preliminary work evaluated the thermodynamic surface characteristics and wettability of NTHP and RMPP on 3DR [33], these surface-energy modifications served only as predictive indicators rather than direct measures of adhesive performance. Within the scope of our literature search, no study has been identified that evaluated the shear bond strength (SBS) and thermal aging resistance of NTHP and RMPP on 3DR fabricated in FLR geometries using an interface-only protocol, or that directly compared their bonding efficacy with conventional APA and UA strategies before and after thermal aging. Therefore, the aim of this study was to evaluate the SBS, artificial thermal aging stability, and failure modes of 3DR specimens in FLR dimensions conditioned with NTHP or RMPP compared to conventional surface treatment protocols.

## 2. Materials and Methods

### 2.1. Specimen Preparation

**Figure 2:**
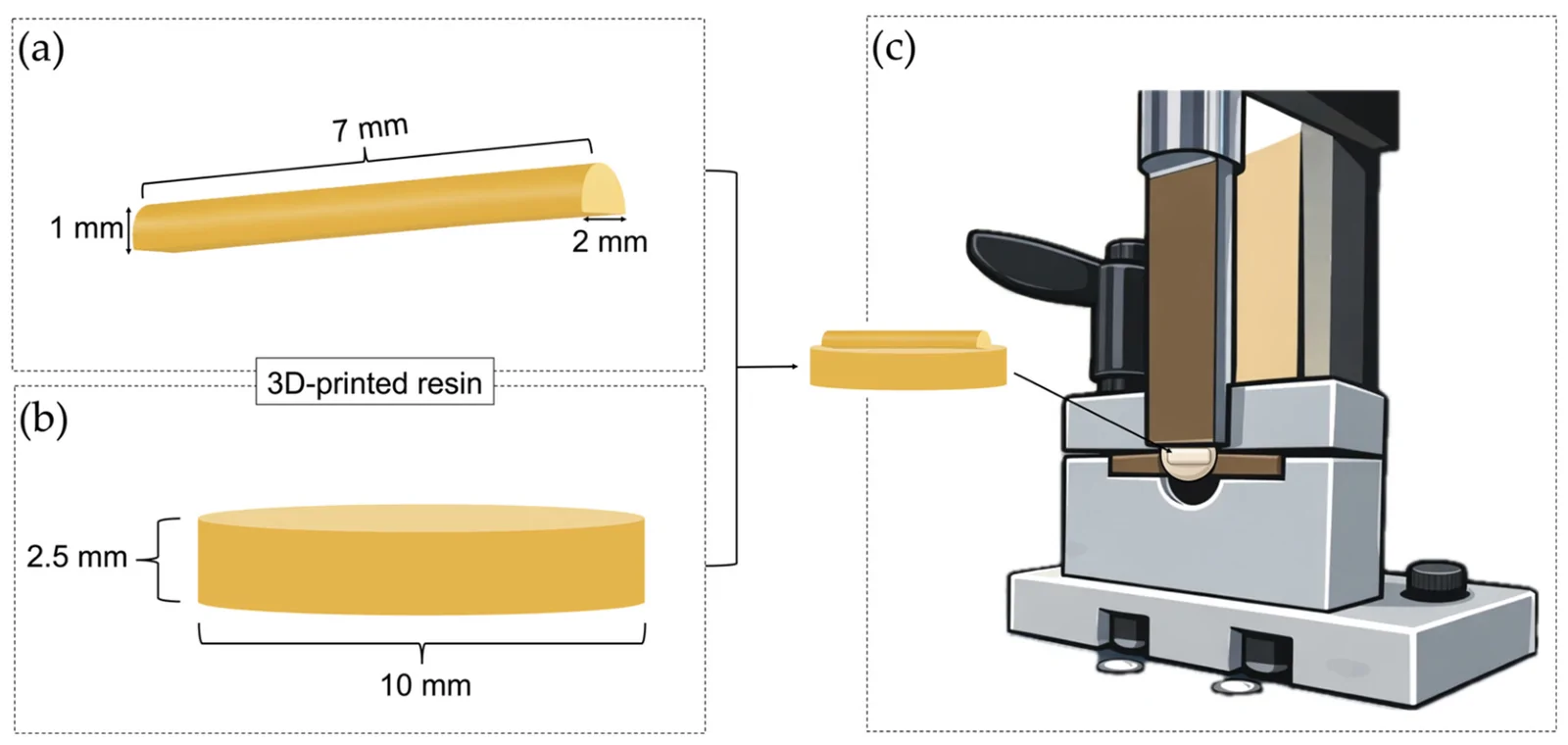
Schematic illustration of specimen geometries and experimental testing configuration: (**a**) hemi-elliptical specimen served as the shear body adherent; (**b**) disc-shaped specimen used for surface characterization and as the bonding substrate; and (**c**) schematic of the shear bond strength testing setup showing load application at the adhesive interface.

Specimens were fabricated using the materials detailed in Table 1 based on designs created in SolidWorks 2022 (Dassault Systems, Waltham, MA, USA) and exported as STL files. An experienced technician printed the samples using a 3Demax printer (DMG Medical Devices, Hamburg, Germany). Printing was conducted under controlled ambient conditions (25 ± 2 °C, 50 ± 10% relative humidity) at a layer thickness of 0.05 mm, an exposure time of 2.5–3.0 s per layer (initial base layers: 15–20 s), and a horizontal build orientation (0° build angle) relative to the build platform. Automatic pillar supports with a 0.8-mm tip diameter were generated for print stabilization. Two geometries were produced (Figure 2a,b): disc-shaped specimens (10-mm diameter × 2.5-mm thickness) allocated for surface roughness, water-contact angle (WCA), attenuated total reflectance-Fourier transform infrared (ATR-FTIR) spectroscopy, and SBS testing, and hemi-elliptical specimens (1-mm thickness, 2-mm width, 7-mm length), utilized specifically for SBS analysis. Both the material selection and the hemi-elliptical dimensions were adopted from our previous study [6], which established this specific material and profile as an optimized design for FLRs owing to its high flexural strength and physiological flexibility. To ensure geometric accuracy, this hemi-elliptical specimen was designed by bisecting a symmetric 2D ellipse along its major axis and linearly extruding the remaining profile to the target length of 7 mm. The overall experimental setup and SBS evaluation methodology were adapted from the previous literature, where a circular base specimen combined with a shear body was utilized to investigate adhesive interfacial bonding on 3DR substrates [34].

Following printing, the specimens were allowed to rest on the build platform for 5 min before removal. They were subsequently cleaned in isopropyl alcohol (IPA) using ultrasonic agitation for 1 min and thoroughly air-dried. Post-curing was carried out in a xenon flash curing unit (Otoflash G171, NK-Optik GmbH, Baierbrunn, Germany) operated at 2 × 2000 flashes under a protective nitrogen gas atmosphere according to the manufacturer’s instructions. Following post-curing, the bonding surfaces of all specimens were ground with 600-grit abrasive paper and ultrasonically cleaned in distilled water (DW) using an ultrasonic bath (KUDOS-SK5200 BTU; Lenon Instruments Co., Ltd., Taoyuan, Taiwan) at 40 kHz for 10 min.

An a priori power analysis was performed using G*Power software (v 3.1.9.7, Heinrich Heine University Düsseldorf, Düsseldorf, Germany) within a one-way ANOVA framework to determine the required sample size for each experimental phase. For the baseline surface characterization assays (surface roughness and WCA) across 5 experimental groups, the effect size (*f* = 0.555) was derived from the previous study, with an α-error of 0.05, and a target power of 0.80. This yielded a minimum sample size of 9 specimens per group; consequently, 10 specimens per group were utilized (total N = 50) [19]. For SBS evaluation across the 10 experimental groups (5 surface treatments × 2 aging conditions), a more conservative effect size of *f* = 0.45 was specified within a factorial design framework (numerator df = 4 for the interaction term), with an α-error of 0.05 and a target power of 0.95 to provide high sensitivity for detecting interaction effects. This yielded a minimum total sample size of 97 specimens; therefore, 11 specimens per group were enrolled (total N = 110).

Surface-treatment procedures were applied to both the disc-shaped and hemi-elliptical specimens. The control group (NT) received no additional surface treatment. For the APA procedure, the specimens were treated using a fine sandblasting unit (BASIC eco; Renfert GmbH, Hilzingen, Germany) with 50-µm Al_2_O_3_ particles at a pressure of 1 bar from 10 mm for 10 s. To ensure protocol reproducibility and eliminate operator-induced variability, a single experienced dental technician performed the procedure using a standard straight nozzle tip (0.8-mm internal diameter) positioned perpendicular (90° nozzle angle) to the substrate surface, delivering a uniform particle flow rate of approximately 1.5–2.0 g/min. A fixed 10-mm visual calibration guide was utilized to strictly maintain the nozzle distance, while a standardized, uniform back-and-forth scanning motion was executed across the specimen surface to guarantee homogenous macro-abrasion. This was followed by a 5-s rinse with DW and subsequent air-drying. For the plasma treatment, a handheld plasma device (PiezoBrush PZ3, Relyon Plasma GmbH, Regensburg, Germany) utilizing ambient air as the process gas was rigidly secured to a laboratory stand to standardize treatment delivery. The device was operated at 18 W and 50 kHz for 30 s at standard room temperature, maintaining a strict vertical orientation (90° impact angle) and a fixed distance of 10 mm directly above the center of the specimen. A 30-s waiting period was observed before the application of the bonding agent. For UA and RMPP, a single coat of the bonding solution was applied to the entire bonding surface using a microbrush, gently air-dried for approximately 5 s, and then light-cured for 10 s in strict accordance with the manufacturers’ instructions. Polymerization was performed using a light-curing unit (Litex 696 Turbo; Dentamerica Inc., City of Industry, CA, USA) operated in regular mode at a wavelength range of 400–500 nm, and an irradiance of 1200 mW/cm^2^. The specimen classifications and their corresponding abbreviations are listed in Table 2.

Specifically, for the SBS test, following the respective surface treatments, the bonding surfaces of the hemi-elliptical specimens were coated with composite resin and positioned centrally on the bonding surfaces of the disc-shaped specimen. To minimize technique variability, a single operator seated each specimen under a standardized load of 4.9 N for 10 s using a custom vertical positioning jig, ensuring uniform adhesive film thickness. Excess resin around the bonding footprint was meticulously removed with a microbrush prior to light polymerization. A light curing unit (Litex 696 Turbo; Dentamerica Inc.) equipped with an 8-mm diameter light-guide tip was operated in regular mode at an emission wavelength range of 400–500 nm. Irradiance was verified prior to testing at 1200 mW/cm^2^ using a digital dental radiometer. During light curing, the tip was positioned directly perpendicular to the bonding interface at a standardized distance of 1 mm, and light polymerization was performed for 40 s to ensure complete conversion across the bonded interface at a standardized distance of 1 mm. The bonded specimens were stored in distilled water at 37 °C for 24 h.

### 2.2. Evaluation of Surface Characteristics

Surface roughness (Ra) was measured using a profilometer (Surftest SJ-210, Mitutoyo, Tokyo, Japan) with a cutoff length of 0.8 mm, evaluation length of 6.4 mm, and speed of 0.5 mm/s. For each specimen, three line-scan measurements were acquired across randomly selected central regions of the bonded surface to avoid edge artifacts. The stylus traversal direction was oriented perpendicular (90°) to the build orientation to systematically cross the additive manufacturing layer lines, ensuring that build-layer striations and surface topography were captured. The three independent roughness readings per specimen were arithmetically averaged to yield a single representative mean Ra value.

ATR-FTIR analysis (Thermo Scientific Nicolet™ iS™ 10 FTIR Spectrometer, Thermo Fisher Scientific, Waltham, MA, USA) was employed to examine the functional groups resulting from the surface treatment; spectra were acquired at a resolution of 2 cm^−1^ with 100 scans over a spectral range of 4000–400 cm^−1^, followed by standard baseline correction. WCA was assessed using a contact-angle goniometer (Phoenix Mini, Surface Electro Optics Co., Ltd., Suwon, Republic of Korea) with DW utilizing a controlled drop volume of 50 μL of deionized water and an equilibration/measurement dwell time of 5 s prior to angle calculation. For each specimen, the WCA was measured five times to minimize variability, and the mean value was recorded as the representative WCA.

### 2.3. Artificial Aging and the SBS Test

The experimental groups and their corresponding thermocycled counterparts were evaluated. Specimens in the non-aged groups were tested immediately, whereas those in the thermocycled groups underwent 10,000 thermal cycles between 5 °C and 55 °C using a custom-built automated thermocycling device with a 30-s dwell time in each bath. For the SBS setup, each bonded specimen was securely mounted into a designated fixing hole within a custom specimen holder, and tightened to ensure rigid stabilization. The bonded hemi-elliptical specimen protruded outward, fully exposed for debonding. This assembly was aligned in the universal testing machine so that a vertical compressive load could be applied directly and parallel to the bonding interface. Subsequently, SBS testing was performed using a universal testing machine (JSV-H1000; Japan Instrumentation System Co., Ltd., Nara, Japan) operating at a crosshead speed of 1 mm/min until failure (Figure 2c). Eccentric loading and localized stress concentrations were minimized by positioning a flat-edged chisel blade (7.0 mm in width) parallel and directly adjacent (<0.2 mm) to the interface line, thereby distributing the shear force uniformly across the entire contact length. This mechanical setup and interface-adjacent blade alignment adhere to established standardized protocols for shear bond testing of indirect resin restorations and CAD/CAM resin-matrix materials [35]. For each specimen, the maximum load at failure was recorded and the SBS was calculated by dividing this maximum load by the exact nominal bonded surface area of 14.0 mm^2^, which corresponds to the rectangular base footprint (2.0 mm width × 7.0 mm length) of the hemi-elliptical specimen bonded to the center of the disc substrate.

### 2.4. Failure-Pattern Evaluation

After the SBS test, the debonded surfaces of the disc specimens were examined using a high-definition video microscope (DMSZ30-C10; Yuyao Dagong Instrument Co., Ltd., Yuyao, China) at 20× and 40× magnifications to determine their failure modes, and assess the representative interfaces.

Failure modes were classified as follows:

- Adhesive failure: Debonding occurred at the interface between the disc-shaped and hemi-elliptical specimens, with both structures remaining intact.
- Cohesive failure: A fracture or visible crack propagates exclusively within the material itself (either the disc-shaped substrate or hemi-elliptical specimen) without involving the bonding interface.
- Mixed failure: Evidence of both interfacial debonding and structural damage to the hemi-elliptical specimen is present. This included cases in which the hemi-elliptical specimen fractured or exhibited a crack line without complete fracture of the disc-shaped specimen.

### 2.5. Statistical Analysis

Statistical analysis was performed using a statistical software program (IBM SPSS statistics, v25.0, IBM Corp., Armonk, NY, USA). Data distribution was assessed using the Shapiro–Wilk test. Continuous parametric variables were presented as mean ± standard deviation (SD), whereas non-normally distributed datasets were reported as both mean ± SD and median [interquartile range, IQR]. For non-normally distributed parameters (surface roughness and water-contact angle), overall differences among the surface-treatment groups were evaluated using the non-parametric Kruskal–Wallis test, followed by Dunn’s post hoc pairwise comparisons.

For the SBS data, a two-way factorial analysis of variance (ANOVA) was performed to evaluate the main effects of surface treatment, aging, and their interaction (surface treatment × aging). When a significant interaction was detected, simple main effects were further analyzed. Differences among the groups within each aging condition (pre-aging and post-aging) were evaluated using one-way ANOVA followed by Tukey’s post hoc test. Because the pre-aging and post-aging specimens were independent, differences between aging conditions within each group were evaluated using the Mann–Whitney U test. Additionally, localized pairwise failure mode distributions (adhesive, cohesive, and mixed) were evaluated; due to the high frequency of cells with low expected counts (<5), statistical significance was determined using the Fisher exact test and Fisher–Freeman–Halton exact test via Monte Carlo simulation. The significance level was set at α = 0.05 for all statistical tests.

## 3. Results

### 3.1. Surface Characteristics

**Figure 3:**
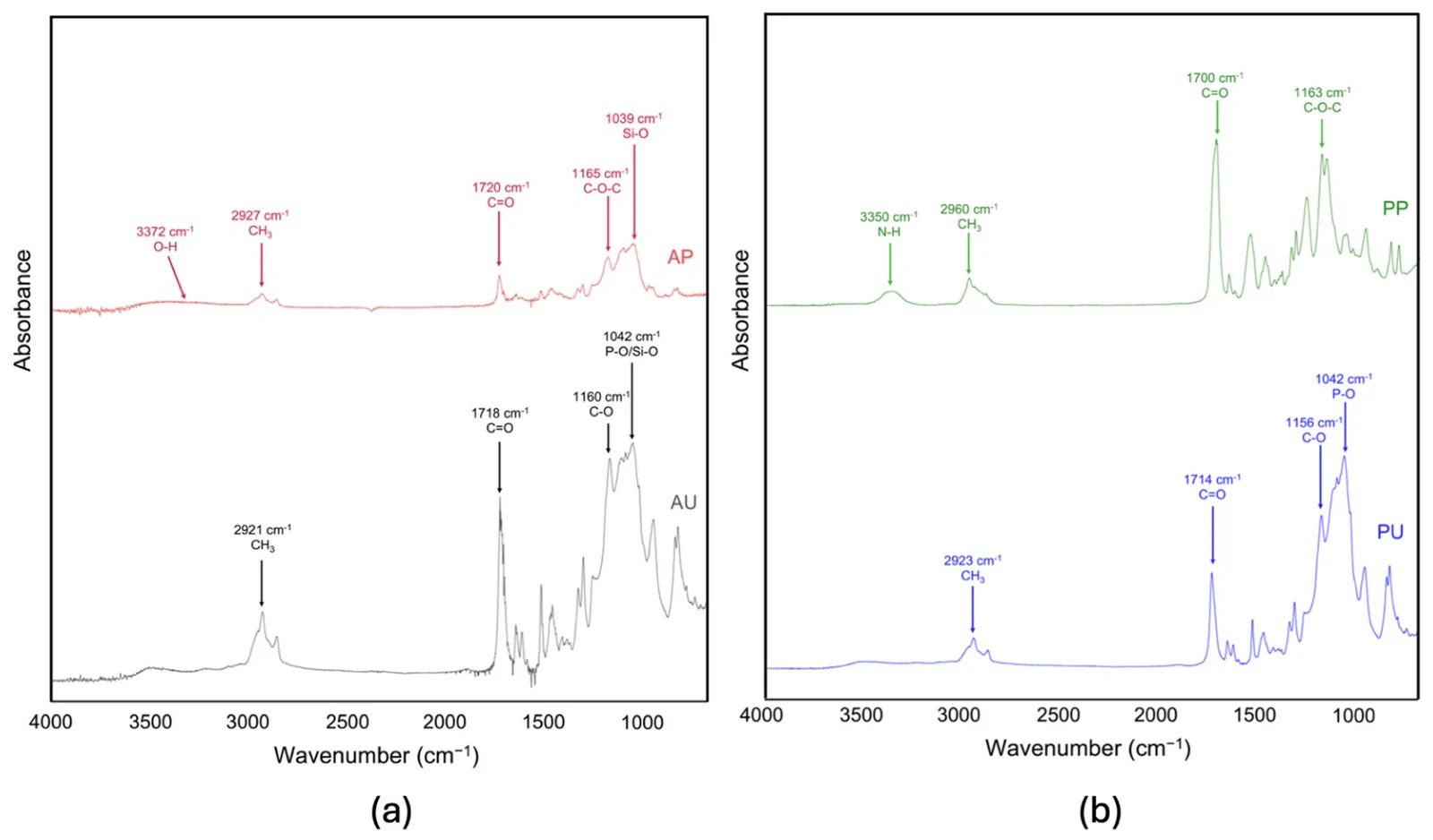
ATR-FTIR analysis of 3D-printed resin specimens following different surface treatments: (**a**) AP—airborne-particle abrasion (APA) followed by resin-matrix-penetrating primer (RMPP) application and AU–APA followed by universal adhesive (UA) application. (**b**) PP—non-thermal handheld plasma (NTHP) treatment followed by RMPP application and PU–NTHP treatment followed by UA application.

ATR-FTIR spectroscopy (Figure 3) revealed classic methacrylate peaks (CH3, C=O, and C–O) in both UA- and RMPP-treated groups. Notably, the PP group exhibited a distinct N–H stretching peak at approximately 3350 cm^−1^, corresponding to urethane dimethacrylate (UDMA). In contrast, the N–H signal in the AP group was masked by a broad O–H stretching band near 3372 cm^−1^, possibly owing to residual water remaining after cleaning and rinsing the APA samples. The P–O stretching band of 10-MDP was observed at approximately 1042 cm^−1^ in both the AU and PU group.

Table 3 shows the mean Ra values across experimental groups. The NT group showed no significant differences compared with the NTHP-treated specimens (*p* > 0.05). In contrast, APA significantly increased surface roughness relative to the NT group (*p* < 0.05). Furthermore, the APA-treated groups (AU and AP) exhibited significantly higher roughness than those treated with NTHP (PU and PP) (*p* < 0.05). Although the roughness was lower following RMPP treatment compared to the corresponding UA applications, these differences were not statistically significant (*p* > 0.05).

Table 3 also presents the mean WCA values across all experimental groups, with the NT group exhibiting the highest hydrophobicity at 95.3 ± 9.8°. Following surface treatment, all groups showed a significant increase in wettability compared to the NT group (*p* < 0.05). Notably, the UA-treated groups (AU and PU) demonstrated the highest hydrophilicity, with contact angles decreasing significantly to 15.4 ± 2.5° and 17.3 ± 2.5°, respectively; no significant difference was observed between the AU and PU groups (*p* > 0.05). In contrast, the RMPP-treated groups (AP and PP) exhibited intermediate wettability, with no significant difference observed between them (*p* > 0.05). Ultimately, while surface roughness was primarily governed by the underlying mechanical pretreatments, the WCA was predominantly driven by the different chemical treatments applied.

### 3.2. Bonding Performance

**Figure 4:**
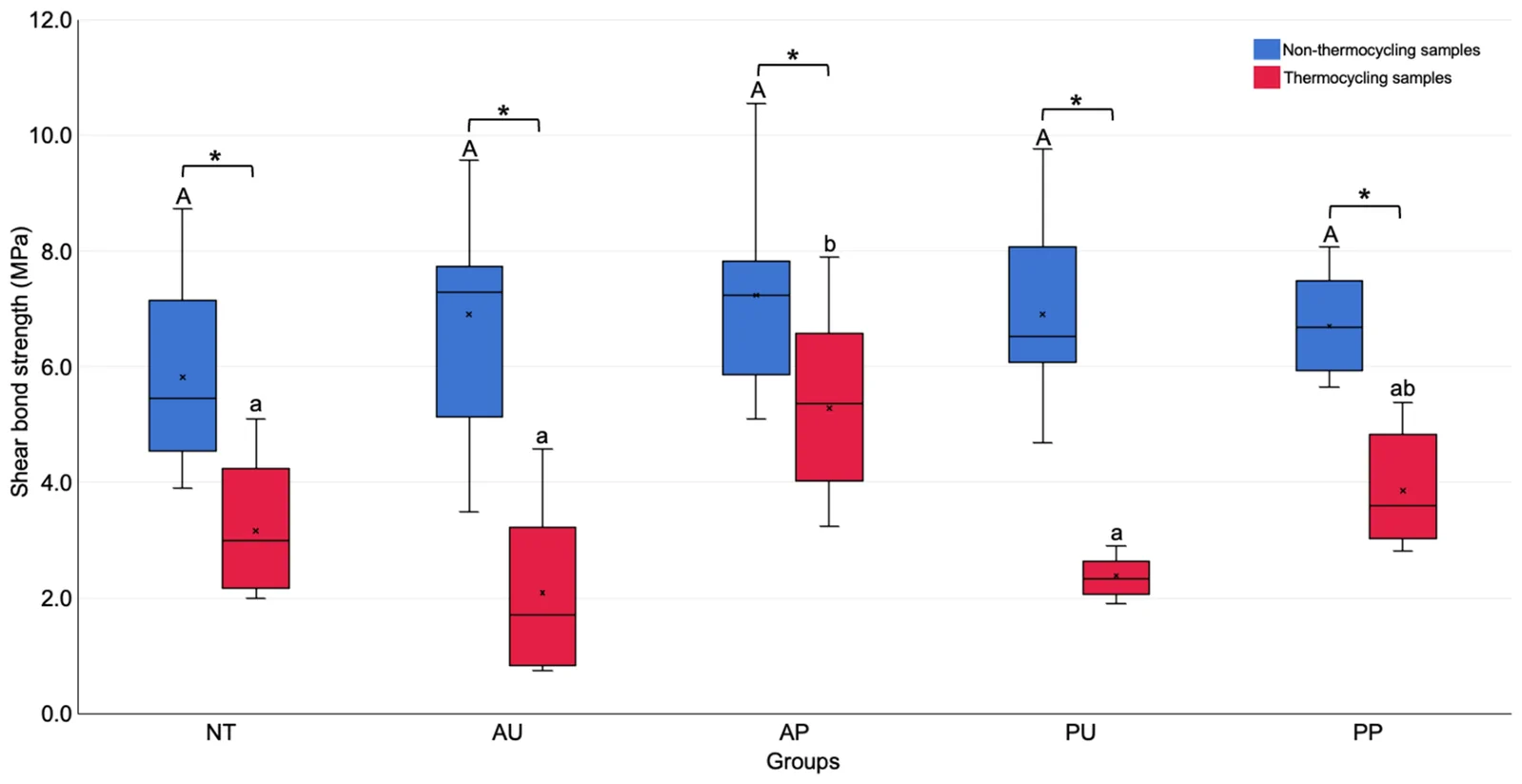
Shear bond strength of 3D-printed resin specimens subjected to different surface treatments before and after thermocycling. Horizontal lines within boxes denote medians, “×” symbols represent means, box boundaries define the interquartile range (25th–75th percentiles), and whiskers extend to minimum and maximum values. An asterisk (*) denotes statistically significant differences between the paired pre- and post-thermocycling groups (*p* < 0.05). For comparisons among different surface treatments within the same aging condition, identical uppercase letters (all sharing “A”) indicate that there are no statistically significant differences among the non-thermocycling groups (*p* ≥ 0.05), whereas distinct lowercase letters (e.g., a, b, ab) denote statistically significant differences among the thermocycling groups (*p* < 0.05).

Figure 4 shows the SBS values of the 3DR specimens after the application of various surface treatments. A two-way factorial ANOVA revealed significant main effects for both surface treatment (F_4,100_ = 7.101, *p* < 0.001, $\eta_{p}^{2}$ = 0.221) and aging (F_1,100_ = 175.033, *p* < 0.001, $\eta_{p}^{2}$ = 0.636). More importantly, a statistically significant interaction effect was observed between surface treatment and aging (F_4,100_ = 4.742, *p* = 0.002, $\eta_{p}^{2}\;$= 0.159), confirming that bond degradation behavior over the aging timeline was directly dictated by the specific surface treatment applied.

Consequently, simple main effects were analyzed to break down these interaction dynamics. Prior to artificial aging, one-way ANOVA indicated no statistically significant differences in SBS values among the experimental groups, irrespective of surface pretreatment. However, artificial aging significantly compromised interfacial integrity across all cohorts, with each group exhibiting a significant reduction in SBS ranging from 26.9% to 69.8% relative to its non-aged counterpart (Mann–Whitney U test, *p* < 0.01). Despite this general decline, simple main effect comparisons within the post-aging state demonstrated that the AP group preserved a mean SBS of 5.3 ± 1.5 MPa after thermocycling, which was significantly higher than the values observed in the NT group (3.2 ± 1.1 MPa) and the groups utilizing UA (AU: 2.1 ± 1.4 MPa and PU: 2.4 ± 0.4 MPa). The intergroup comparisons showed that NT, AU, PU, and PP were not significantly different from one another, and PP (3.8 ± 0.8 MPa) did not differ from any group. Only the AP group exhibited statistically superior bond durability compared to NT, AU, and PU, while remaining statistically comparable to PP.

**Figure 5:**
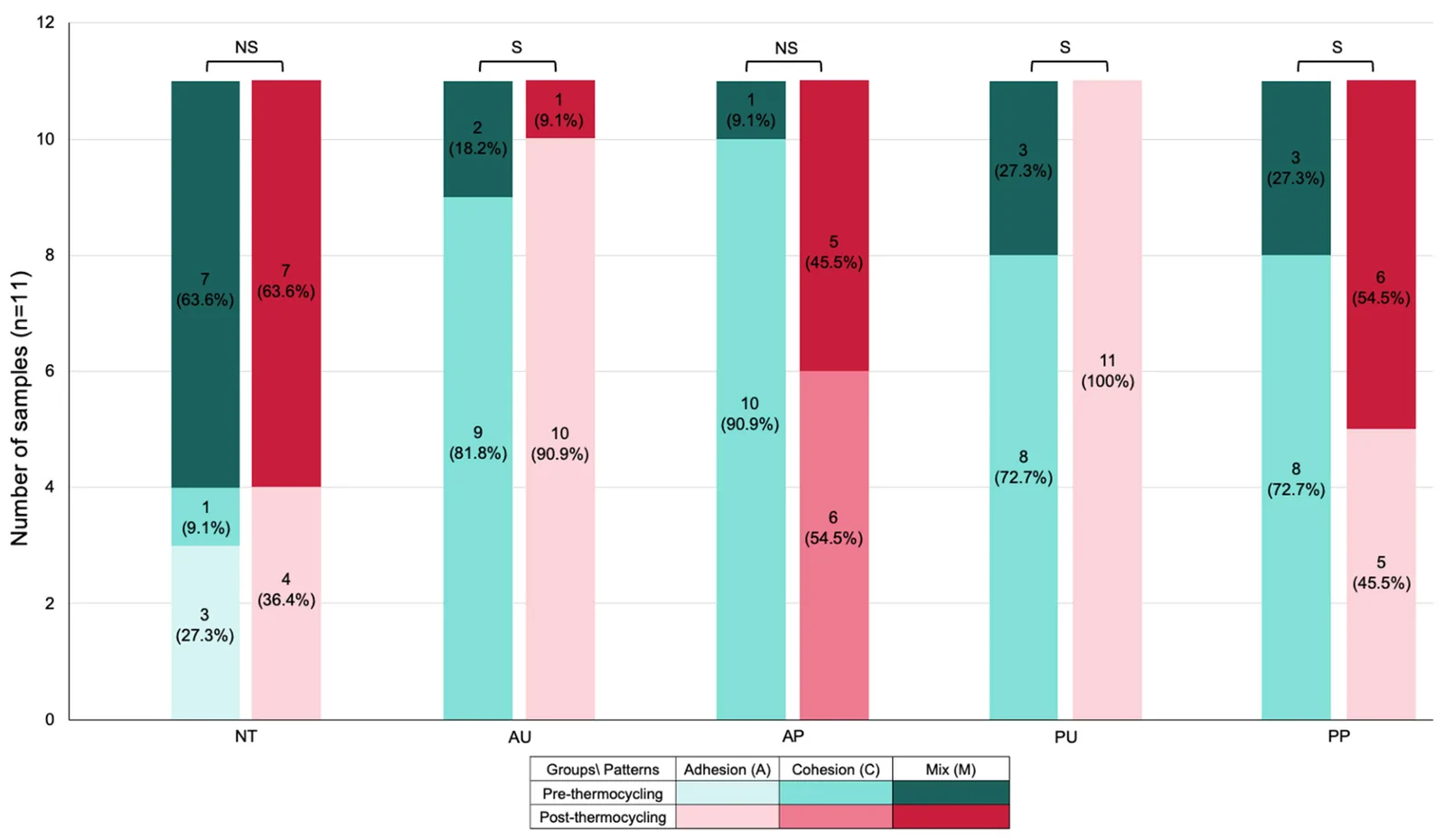
Distribution of fracture patterns of 3D-printed resin specimens across different surface treatments before and after thermocycling. ‘S’ denotes a statistically significant difference between pre- and post-thermocycling failure mode distributions within the same surface treatment group (*p* < 0.05), while ‘NS’ denotes no statistically significant difference (*p* ≥ 0.05) based on the Fisher–Freeman–Halton exact test.

**Figure 6:**
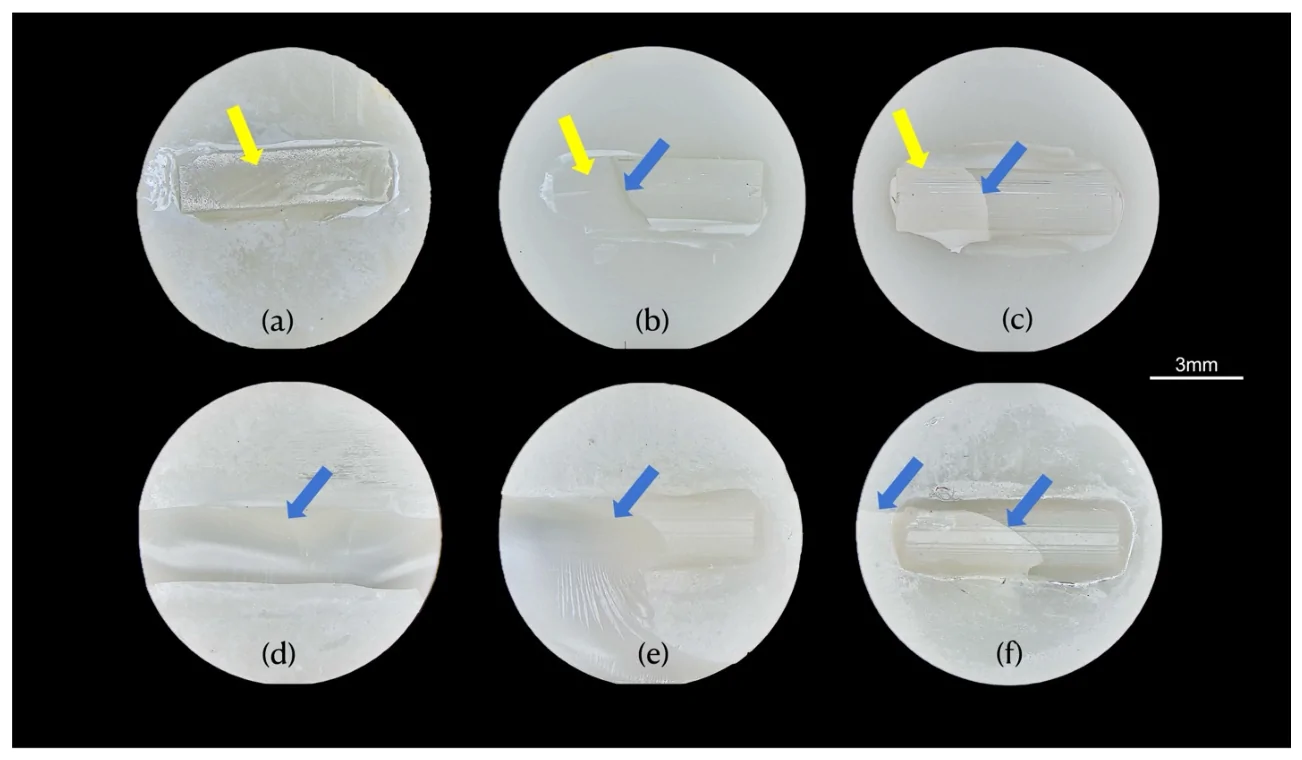
High-definition video microscopic examination of representative debonded interfaces and fracture patterns at 20× effective magnification. Images reveal: (**a**) adhesive failure; (**b**,**c**) mixed failure; and (**d**–**f**) cohesive failure where yellow arrows indicate failure at the bonding interface and blue arrows indicate failure within the 3D-printed resin specimens.

The distribution of failure patterns for each group and representative failure-mode images is presented in Figure 5 and Figure 6, respectively. Before thermocycling, all the surface-treated groups (AU, PU, AP, and PP) exhibited a predominance of cohesive failure, whereas the NT group primarily exhibited mixed failure. After aging, the AU and PU groups showed the most dramatic loss of bond integrity, and shifted from mostly cohesive to almost entirely adhesive failure (90.9% and 100%, respectively). The Fisher–Freeman–Halton exact test confirmed that these temporal shifts in fracture mechanics were highly statistically significant for both the AU group (*Χ*^2^ = 19.333, *p* < 0.001) and the PU group (*Χ*^2^ = 22.000, *p* < 0.001), demonstrating a critical, aging-induced degradation of the adhesive interfaces over time. In contrast, the AP group maintained superior interface stability, remaining primarily cohesive (54.5%) and mixed (45.5%) without adhesive failure. Fisher’s exact test confirmed that this minor distribution shift was not statistically significant (*Χ*^2^ = 3.667, *p* = 0.149), indicating that the structural integrity of the bonding joint was successfully preserved despite the aggressive thermal aging process. The PP group showed a marked shift toward the interface—transitioning from cohesive failure (72.7%) to a mixture of mixed (54.5%) and adhesive (45.5%) patterns. The Fisher–Freeman–Halton exact test confirmed that this transition in fracture characteristics over the aging timeline was statistically significant (*Χ*^2^ = 14.000, *p* < 0.001). Notably, the NT group remained stable, maintaining its primary mixed failure mode, both before and after thermocycling. The analysis verified that this baseline stability was statistically supported, showing absolutely no significant alteration in failure characteristics following environmental aging (*Χ*^2^ = 1.143, *p* = 1.000).

## 4. Discussion

Chemical interactions at the adhesive interface were characterized by identifying methacrylate absorption bands across all groups, which confirmed the presence of the resin matrix. The PP group showed distinct C–O–C stretching (at ~1163 cm^−1^), C=O carbonyl stretching (at ~1700 cm^−1^), CH_3_ bending (at ~2960 cm^−1^), and N–H bending (at ~3350 cm^−1^), confirming the presence of characteristic functional resin groups on the conditioned surface [36,37]. In the AP group, the N–H stretching band of UDMA was masked by a broad O–H absorption at 3372 cm^−1^, likely owing to residual moisture from post-abrasive cleaning. Notably, the distinct Si–O stretching vibration detected at ~1039 cm^−1^ indicates the exposure of the inorganic silica fillers within the 3DR matrix. This is consistent with the effective removal of the superficial organic matrix by APA and thereby unmasks the underlying silica phase. This observation aligns with the findings that Si–O-related signals in adhesive systems are frequently masked by overlapping organic peaks but can become detectable when surface treatments reduce such interference [38]. For the UA-treated groups, the characteristic methacrylate peaks and C–O stretching bands at 1160 cm^−1^ (AU) and 1156 cm^−1^ (PU) confirmed the presence of 10-MDP [38]. Moreover, the distinct signal of the AU group at 1039 cm^−1^ was attributed to Si–O stretching from the exposed silica filler overlapping with P–O stretching from 10-MDP. In the PU group, 1042 cm^−1^ corresponded primarily to P–O stretching of the 10-MDP monomer, as NTHP treats the superficial layer without exposing underlying structural filler particles [38].

The RMPP groups exhibited lower surface roughness than the corresponding UA groups did, whereas NTHP and UA treatments did not significantly alter roughness, considering the lack of significant differences between the NT and PU groups. Although a previous study utilizing low-pressure plasma demonstrated that it could generate a surface roughness comparable to APA, our results showed that NTHP did not significantly modify the surface topography [39,40]. The lower surface roughness observed in the RMPP groups may be related to the viscosity and film-forming characteristics of its constituent monomers, where high-mobility low-viscosity MMA could facilitate infiltration into micro-irregularities while the more viscous UDMA acts to smooth surface topography prior to light curing [41]. This creates a smoother transition layer compared to the UA groups, where the film thickness of one-step UA is frequently <10 µm and the air-thinning to evaporate solvents further reduces the volume of the adhesive and thereby prevents it from effectively covering all the surface irregularities [42].

The NT group exhibited a WCA of 95.3 ± 9.8°, which is comparable to values previously reported for permanent resin crown materials used as resin posts (85.2 ± 2.0°) [43]; minor variations are likely attributed to differences in material compositions. Notably, the UA-treated groups (AU and PU) demonstrated the highest hydrophilicity, with contact angles decreasing significantly to 15.4° and 17.3°, respectively. This is primarily owing to the inclusion of 2-hydroxyethyl methacrylate (HEMA), which is a hydrophilic monomer that reduces surface tension and contact angle. This enhanced surface adaptation promotes monomer infiltration into the microtopography of the resin [24]. In contrast, the RMPP-treated groups exhibited intermediate wettability compared to the NT group, likely because of the formation of a continuous, resin-rich surface layer with elevated surface energy following the infiltration and polymerization of MMA- and UDMA-containing monomers [44].

No statistically significant differences in SBS were detected among the experimental groups, regardless of surface pretreatment. This finding is consistent with previous research on permanent 3DR materials that reported comparable SBS across different surface treatments before thermocycling [20]. Variations in SBS values may be attributed to differences in 3D-printed material brands, shear body design, and methodological parameters, such as APA pressure and resin cement. To contextualize the baseline adhesive capacity of these 3DR assemblies, the observed SBS values were benchmarked against the established range of 5.9–7.8 MPa for orthodontic bracket retention [45]. Notably, the pre-aging SBS values of all experimental groups fell within or approached this threshold limit: the NT, AU, AP, PU, and PP groups were at 5.8 ± 1.7, 6.9 ± 2.0, 7.2 ± 1.5, 6.9 ± 1.4, and 6.8 ± 1.1 MPa, respectively. Although the AP group exhibited a numerically higher mean SBS than the NT group, post-hoc analysis confirmed that this difference was not statistically significant (*p* = 0.533), supporting the conclusion that no significant differences existed among the pre-aging SBS values. The hemi-elliptical shear body design was used in this study based on its favorable flexural strength and suitability for the FLR [6]. Previous studies have revealed that a hemi-elliptical profile optimizes mechanical strength, flexibility, patient comfort, and debonding force resistance [16,46,47]. Biomechanically, this geometry supports a minimalist, streamlined retainer design that completely eliminates the need for a traditional encapsulation method (or) bulky retention pad. Because the profile lacks sharp angles, mechanical stresses are deflected along its smooth, curved surface, reducing stress concentration and enhancing patient comfort. Concurrently, this hemi-elliptical shape provides a flat, widened base that maximizes the direct bonding surface area. This architecture allows a true ‘interface-only’ retention mechanism to be achieved, ensuring the laboratory model evaluates the interfacial bonding potential of a scaled-down, streamlined retainer. These SBS values indicate that even with a streamlined, interface-only design, the 3DR can provide sufficient initial adhesion for clinical applications. The Reynolds benchmark [45] was derived from metallic bracket bases bonded to natural enamel, which involve distinct surface compliance, adhesive interfaces, and complex masticatory force vectors compared to a polymer-to-polymer assembly. Within the standardized parameters of this laboratory setup, these pre-aging values simply demonstrate that an interface-only geometry can achieve initial bonding levels comparable to standard orthodontic attachment thresholds. APA was performed at 1 bar because higher pressures do not provide additional SBS benefits and may increase the risk of substrate damage [34].

Failure-mode analysis revealed predominantly mixed failures in the NT group (63.6%), whereas surface-treated groups showed a higher incidence of cohesive failures, including AU (81.8%), AP (90.9%), PU (72.7%), and PP (72.7%). This shift indicates that failure was primarily governed by the material strength rather than the interfacial bond integrity. Although the NT group exhibited a substantial proportion of mixed failure (63.6%), the presence of adhesive failure (9.1%) suggested a more interface-dependent bonding behavior with reduced stress transfer into the substrate. Cohesive and mixed failure modes are generally considered more favorable than adhesive failure because the latter is typically associated with a lower bond strength [48,49]. Moreover, no adhesive failure was observed in the surface-treated groups, which exclusively exhibited cohesive or mixed failures. Specifically, APA produced a high rate of cohesive failure (AU: 81.8% and AP: 90.9%), likely owing to enhanced micromechanical retention from surface roughening, consistent with previous findings [50]. Moreover, the NTHP-treated groups demonstrated high cohesive failure rates accompanied by mixed failures, suggesting a balanced enhancement of surface chemical reactivity and micromechanical features.

The SBS decreased significantly in all groups after thermocycling. Crucially, the implementation of a two-way factorial ANOVA substantiated a statistically significant interaction effect between surface treatment and thermocycling aging (F_4,100_ = 4.742, *p* = 0.002). This interaction confirms that the rate and severity of interfacial bond degradation were fundamentally governed by the specific surface conditioning applied, rather than representing a uniform decline across all groups. Consequently, examining the simple main effects reveals that while baseline bonding efficacy was universally acceptable, hydrothermal fatigue actively discriminated between stable and unstable bonding interfaces. Despite the general decline in strength, the AP group maintained a mean SBS of 5.3 ± 1.5 MPa after thermocycling, which is significantly higher than the values seen in NT (3.2 ± 1.1 MPa) and groups utilizing UA (AU: 2.1 ± 1.4 MPa and PU: 2.4 ± 0.4 MPa). The intergroup comparisons showed that NT, AU, PU, and PP were not significantly different from one another, and PP (3.8 ± 0.8 MPa) did not differ from any group. Only the AP group exhibited significantly different SBS values, and this difference was not observed when compared to the PP group. While these aged values are slightly below the immediate, non-aged 5.9–7.8 MPa bracket benchmark, they should not be interpreted as definitive clinical inadequacy. Unlike orthodontic brackets that must actively resist complex continuous mechanics from archwires, a passive FLR functions primarily to maintain tooth position. Furthermore, digital fabrication allows 3D-printed FLRs to be customized and positioned outside primary occlusal contacts on the lingual fossae, substantially reducing functional loading vectors. In such protected, passive environments, the sustained interfacial stability of the AP group indicates superior resistance to artificial hydrothermal stress, though this in vitro endurance cannot be directly equated with long-term intraoral durability.

UA-treated groups exhibited the greatest reduction in SBS after thermocycling, with decreases of 69.8% for AU and 65.6% for PU. Moreover, the predominant failure mode of UA-treated groups shifted from cohesive to mainly adhesive after thermocycling, with AU exhibiting 90.9% adhesive failure and PU reaching 100%, indicating reduced interfacial integrity after aging. The Fisher–Freeman–Halton exact test mathematically substantiated this behavioral shift, confirming that the temporal degradation of the interface was highly statistically significant for both the AU cohort (*Χ*^2^ = 19.333, *p* < 0.001) and the PU cohort (*Χ*^2^ = 22.000, *p* < 0.001). While ATR-FTIR confirmed the presence of characteristic 10-MDP absorption bands, this interface deterioration is hypothesized to stem primarily from the hydrophilic nature of HEMA, which promotes water sorption and accelerates interfacial hydrolytic degradation over time [24]. Although this proposed mechanism is consistent with previous reports on the reduced long-term stability of HEMA-containing adhesives [51], it represents a mechanistic hypothesis based on macro-mechanical shifts rather than direct in situ tracking of chemical bond cleavage at the nanoscale.

Notably, the NT group maintained relatively higher SBS values and predominantly mixed failures, despite no significant differences compared to the UA-treated group. Pairwise contingency analysis verified that the baseline fracture profile of the untreated control remained statistically unaltered by thermal aging (*Χ*^2^ = 1.143, *p* = 1.000). This trend is consistent with previous findings showing the limited benefit of UA application to untreated or APA-treated 3DR surfaces [20].

The lack of improvement in the PP group further suggests that NTHP does not alter the surface topography and that RMPP requires mechanical surface modification for effective interactions. Crucially, because NTHP activation lacks the capacity to induce micro-roughness, this absence of surface topography deprives the resin primer of physical undercuts and anchoring sites, leaving the interface highly vulnerable to hydrolytic degradation during aging. This contrasts with low-pressure plasma treatment which optimizes 3DR adhesion by inducing a “corrosion effect” and creating micropores [41]. After pre-aging, although the PP group exhibited SBS values similar to those of the AP group, the differences were non-significant in the remaining groups. The adhesive failure in the PP group (45.5%) after aging indicated that its bond reliability was not equivalent to that of the AP group (0%). This interface-ward degradation over time was corroborated by the Fisher–Freeman–Halton test, which revealed a highly significant shift in the PP group’s fracture pattern from baseline cohesive characteristics toward an interface-dominated distribution (*Χ*^2^ = 14.000, *p* < 0.001). Overall, the AP group demonstrated the most favorable performance and achieved the highest SBS after thermocycling and superior interfacial stability. Its reliability was supported by the failure mode analysis, which revealed 54.5% cohesive and 45.5% mixed failures, without adhesive failures. Crucially, Fisher’s exact test confirmed that this distribution did not represent a statistically significant alteration from its pre-aged baseline (*Χ*^2^ = 3.667, *p* = 0.149), validating that the chemical and mechanical integrity of the AP junction was effectively locked over time. This marked disparity in degradation resistance—statistically supported by the treatment × aging interaction (*p* = 0.002)—underscores that the combination of mechanical micro-retention (APA) and chemical monomer infiltration (RMPP) forms an optimized synergy capable of resisting thermal and hydrolytic fatigue that destabilizes universal adhesive or untreated interfaces. The sustained bonding performance after thermocycling suggests a potential synergy within this dual-bonding mechanism: APA generates the necessary micro-retentive topography, while low-molecular-weight MMA monomers in RMPP are suggested to infiltrate surface micro-irregularities and interact with the resin matrix, which may help preserve interfacial integrity during thermal aging [30,31]. This proposed interpenetration and interfacial interaction model is consistent with our empirical findings, namely sustained post-aging SBS, the absence of adhesive failures, and the presence of distinct UDMA/methacrylate-related peaks in ATR-FTIR. However, these findings do not directly demonstrate monomer infiltration or interfacial co-polymerization; therefore, the exact sub-micron depth of monomer diffusion and the nature and extent of interfacial chemical interaction remain theoretical explanations requiring future validation through advanced nanoscale characterization. Although the initial SBS values were comparable across all the surface-treatment groups, the APA-treated specimens exhibited a higher frequency of cohesive failure before aging. The occurrence of fractures in the thicker disc-shaped specimen suggests a reduction in the structural integrity of the material. From a structural mechanics perspective, while high surface roughness from APA enhances mechanical interlocking, it inherently introduces sharp surface micro-grooves that act as localized stress concentrators. For thin AM parts, such as low-profile FLRs, these micro-defects can significantly lower the threshold for cyclic fatigue propagation under repetitive masticatory forces. However, a major clinical advantage of utilizing 3DRs for FLRs is the ability to customize and digitally position the appliance entirely out of the patient’s occlusion. By designing the retainer geometry to safely bypass direct masticatory forces, the clinical risk of load-induced fatigue failure at these micro-grooves is naturally minimized in vivo. Consequently, the benefits of mechanical interlocking at the adhesive interface heavily outweigh the slight compromise in bulk material strength under the parameters of this study.

Crucially, the favorable bonding stability demonstrated by this optimized surface engineering protocol (APA–RMPP) highlights the feasibility of minimalist AM designs under laboratory aging conditions. By demonstrating that a targeted interface-only protocol can sustain high bond strengths without relying on bulky resin encapsulation or oversized retention pads, these findings establish design rules for truly minimalist clinical geometries. Eliminating these redundant structural features significantly reduces post-print material volume, lowering both raw resin consumption and subsequent post-processing waste. More broadly, the results support hybrid manufacturing workflows in which AM is integrated with selective surface engineering steps to achieve enhanced interfacial performance while preserving the geometric and material efficiency of AM-produced components.

This study had some limitations. As an in vitro investigation, it cannot replicate all conditions of the oral environment. Furthermore, the specimens were printed as flat experimental blocks rather than actual dental arch-form FLRs. Importantly, this study utilized a 3DR–resin composite–3DR assembly to isolate interfacial bonding behavior rather than testing direct adhesion to tooth enamel, which does not fully represent the clinical retainer-to-enamel interface. Specifically, the evaluation was restricted to a relatively limited sample size per subgroup, a single AM printer system utilizing a single material brand (Detax Freeprint Crown) and a single resin composite cement, which limits immediate generalization across different printing technologies. Crucially, the interface was not subjected to long-term hydrolytic or enzymatic aging—such as exposure to salivary enzymes—nor did it undergo dynamic cyclic loading, which are highly relevant to the clinical lifespan of fixed retainers. Additionally, operator blinding and allocation concealment were not implemented during specimen preparation, SBS testing, or failure mode assessment, which may have introduced potential operator or assessment bias. Moreover, while the failure modes and representative interfaces were systematically examined using a high-definition microscope at varying magnifications, this optical setup provides a reliable macro-level overview of the fracture patterns (adhesive, cohesive, and mixed). It lacks the ultra-high topographic resolution of scanning electron microscopy (SEM), which could further map micro-structural defects or localized interfacial stress propagation. Furthermore, this study did not utilize nanoscale depth profiling, cross-sectional spectroscopy, or nanoindentation to directly quantify monomer penetration depth or verify interfacial chemical degradation mechanisms. To enhance the clinical translatability, future AM-focused research should incorporate larger sample sizes, multi-material and multi-printer validation setups, actual dental arch-form geometries, nanoscale cross-sectional characterization, high-resolution SEM evaluation, long-term enzymatic degradation models, and dynamic cyclic fatigue testing.

## 5. Conclusions

Within the limitations of this in vitro study, the following conclusions were drawn:

1. APA significantly increased substrate surface roughness and created micromechanical retentive features, whereas portable NTHP produced no measurable improvements in surface topography or bond strength under the tested conditions.
2. UA induced the highest hydrophilicity owing to its high HEMA content, followed by RMPP, which formed a hydrophilic resin-rich layer. Notably, the lack of a significant difference between APA and NTHP when paired with the same chemical treatment suggests that chemical treatments masked the underlying mechanical pretreatment.
3. Under in vitro thermocycling conditions, the combination of APA and RMPP may improve the bonding durability of interface-only FLRs; however, clinical viability requires further validation under dynamic intraoral conditions.

## Figures and Tables

**Table 1:** List of materials used.

Material Category	Trade Name (Manufacturer)	Main Components and Characteristics
3D-printed resin (3DR)	Detax Freeprint Crown (Shade A2; Batch No. 260303; Detax GmbH & Co.KG, Ettlingen, Germany)	Multifunctional methacrylate-based resin (alkoxylated phenol methacrylate derivative, 1,6-hexanediol dimethacrylate, bismethacrylate, photoinitiators, pigments)
Air-borne particle abrasion (APA) media	Cobra 50 µm (Renfert GmbH, Hilzingen, Germany)	Al_2_O_3_ particles
Universal adhesive (UA)	3M ESPE Single Bond Universal (Batch No. 41212A; 3M ESPE, Seefeld, Germany)	10-MDP, dimethacrylate resins, HEMA, silane, filler, ethanol, photoinitiators
Resin-matrix-penetrating primer (RMPP)	HC primer (Batch No. 072310; SHOFU Inc., Kyoto, Japan)	UDMA, MMA, solvents, initiators
Composite resin	3M Filtek Supreme Flowable Restorative (Shade A1; Batch No. 7032A1; 3M ESPE, Seefeld, Germany)	Bis-GMA, TEGDMA, Procrylate, filler, photoinitiators

Details of the compositions were obtained from the manufacturer’s safety datasheets. Al_2_O_3_, aluminum oxide; Bis-GMA, bisphenol A–glycidyl methacrylate; HEMA, 2-hydroxyethyl methacrylate; MDP, methacryloyloxydecyl dihydrogen phosphate; MMA, methyl methacrylate; TEGDMA, triethylene glycol dimethacrylate; UDMA, urethane dimethacrylate.

**Table 2:** Group classification and corresponding abbreviations for 3D-printed resins subjected to various surface treatments.

Abbreviations	Groups
NT	Control group with no surface treatment
AU	Airborne-particle abrasion, followed by universal adhesive application
AP	Airborne-particle abrasion, followed by resin-matrix-penetrating primer application
PU	Non-thermal plasma treatment, followed by universal adhesive application
PP	Non-thermal plasma treatment, followed by resin-matrix-penetrating primer application

**Table 3:** Surface roughness and water-contact angle of 3D-printed resin after different surface treatments.

Groups	Roughness (µm)		Water-Contact Angle (Degree)	
	Mean ± SD	Median [IQR]	Mean ± SD	Median [IQR]
NT	0.9 ± 0.1	0.8 [0.1] ^A^	95.3 ± 9.8	96.5 [11.6] ^a^
AU	2.8 ± 0.2	2.8 [0.3] ^B^	15.4 ± 2.5	15.8 [3.9] ^b^
AP	2.0 ± 0.4	1.9 [0.2] ^B^	49.2 ± 2.6	48.4 [3.4] ^c^
PU	0.8 ± 0.3	0.8 [0.5] ^A^	17.3 ± 2.5	18.0 [3.9] ^b^
PP	0.8 ± 0.5	0.8 [0.6] ^A^	50.5 ± 1.2	50.5 [2.1] ^c^

Values are expressed as mean ± standard deviation (SD) and median [interquartile range, IQR]. Within each parameter, values with different superscript letters indicate statistically significant differences (*p* < 0.05), determined by the Kruskal–Wallis test followed by Dunn’s post hoc pairwise comparisons. Uppercase letters denote comparisons for roughness, whereas lowercase letters denote comparisons for water-contact angle. No statistical comparisons were performed between different parameters.

## Data Availability

The original contributions presented in this study are included in the article. Further inquiries can be directed to the corresponding authors.
